# Development of canine C-reactive protein assays

**DOI:** 10.1186/s13028-020-00549-9

**Published:** 2020-09-07

**Authors:** Takaki Waritani, Dawn Cutler, Jessica Chang

**Affiliations:** Chondrex, Inc., 2607 151st Place North East Redmond, Washington, 98052 USA

**Keywords:** C-reactive protein, Dogs, Enzyme-linked immunosorbent assay, Immunochromatography assay, Inflammation

## Abstract

C-reactive protein (CRP), which is released during tissue damage and inflammation, is a useful nonspecific inflammatory marker in both human and veterinary clinical practice. Veterinarians have often used human CRP assays to analyze samples from canine patients, but cross-reactivities between the species affect assay sensitivity and reliability, leading to inaccurate inflammation assessment. To improve the efficiency of inflammation assessment, we developed a canine CRP detection enzyme-linked immunosorbent assay (ELISA) for quantitative analysis and an immunochromatography assay (ICA) for semiquantitative point-of-care (POC) analysis. The ELISA demonstrated an assay detection limit of 0.5 ng/mL, quantitative linear assay range of 1.6–100 ng/mL, and intra- and inter-assay coefficient of variations of 0.7 to 10.0% and 6.0 to 9.0%, respectively; the recovery rates of samples spiked with purified canine CRP were 105 to 109%, and the parallelism assessments were 82.7 to 104.4%. The correlation between the CRP level results obtained with the ELISA and those of a currently available quantitative POC assay was 0.907 with the regression formula of y = 0.55x + 0.05. In addition, the ICA requires only 5 μL samples and a 10-min assay time, and clearly distinguished positive, weak positive, and negative samples (P < 0.001) at an approximately 5–10 µg/mL cut-off value. The developed canine CRP ELISA and ICA showed reliable assay results and a high correlation with a commercially available POC assay in clinical use. The ICA can be a useful canine CRP screening test for diagnostic purposes in veterinary clinics.

## Findings

The level of canine serum C-reactive protein (CRP), an acute-phase protein released during tissue damage and inflammation, is elevated 100- to 1000-fold in surgical trauma, irritation, and inflammatory diseases, including pyometra, panniculitis, acute pancreatitis, polyarthritis, septic arthritis, and hemangiosarcoma [1–3]. The serum level dynamics indicate that canine CRP is a useful, nonspecific, sensitive inflammatory marker [1, 2, 4].

The canine serum CRP level is generally measured with human diagnostic CRP immunoassays, including automated biochemical analyzers [5], enzyme-linked immunosorbent assays (ELISAs) [6, 7] and latex agglutination tests [3, 8]. However, due to the poor cross-reactivity of anti-human CRP antibodies with canine CRP, some of these human CRP assays demonstrate relatively unreliable assay results and mislead inflammation assessment. To achieve more reliable results, several commercial canine CRP-specific assays have been introduced to the market. However, technical improvements are needed to decrease between-run imprecision [9, 10].

Canine CRP ELISAs can be useful for routine checkups but are not appropriate as a rapid test for daily clinical use because of the time and labor required. Recently, commercially available canine CRP point-of-care (POC) assay kits, including quantitative immunoassays using analyzers [11, 12] and semiqualitative immunochromatography assays (ICAs) [10], have been developed. Unfortunately, these kits may still require investigation as they tend to yield imprecise results [13].

The aim of this study was to develop a highly reliable canine CRP ELISA and ICA using anti-canine CRP monoclonal antibodies (mAbs). These assays were validated using clinical samples and compared with a commercially available POC assay (Laser CRP-2 Analyzer from Arrows Co., Ltd, Osaka, Japan) (Laser).

MAbs against canine CRP were developed by cell hybridization of SP2/0-Ag4 cells and splenocytes from BALB/c mice (Envigo, Indianapolis, IN, USA) immunized with purified canine CRP (LEE Biosolutions, Maryland Heights, MO, USA). Hybridomas producing anti-CRP mAbs were screened using an indirect canine CRP ELISA and then cloned by the limiting dilution method. The specificity of the anti-canine CRP mAbs was confirmed by western blot analysis (data not shown). The mAbs produced by hybridomas were purified by Protein G affinity chromatography (GE Healthcare Systems, Chicago, IL, USA) from culture medium collected with a miniPERM culture system (Sarstedt, Newton, SC, USA).

We developed a canine CRP ELISA employing a one-step sandwich assay system using purified canine CRP and two anti-canine CRP mAbs. Briefly, the mAb 4D3C1 was diluted in 100 μL of 0.05 M phosphate-buffered saline buffer, pH 7.4 (PBS), added to each well of a 96-well ELISA plate and incubated at 4 °C overnight. After washing the plates with PBS containing 0.05% Tween 20 (PBST), 50 μL of the mAb 3B4D3 conjugated with horseradish peroxidase in PBS containing 1% bovine serum albumin (BSA) (BSA/PBS) and 50 μL of canine CRP standards or diluted canine serum samples (1:1000 or higher dilution) in BSA/PBS were added to the wells. The plate was incubated for 1 h at room temperature (i.e. around 25 °C). After washing the plates with PBST, color was developed at room temperature for 25 min by adding 100 μL of 3,3′,5,5′-tetramethylbenzidine solution (MP Biomedicals, Santa Ana, CA, USA). After adding 50 μL of 2 N sulfuric acid to stop the enzymatic reaction, absorbance values were measured at 450 nm. The CRP levels in samples were calculated using linear regression based on a standard curve in Microsoft Excel and multiplied by the corresponding dilution factor to obtain true values (μg/mL).

**Fig. 1:**
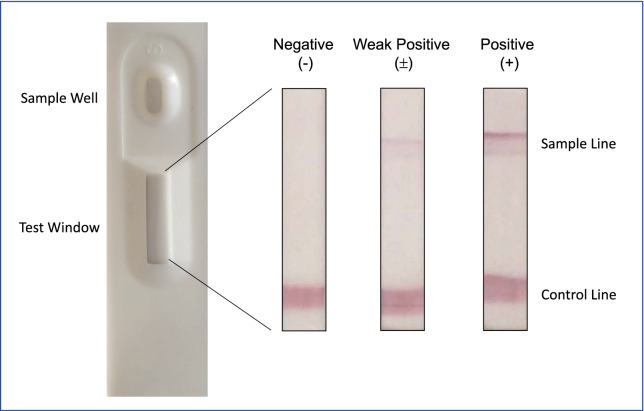
Assay results for the immunochromatography assay. Undiluted canine serum (5 µL) and 100 μL of BSA/PBS containing 0.1% Tween 20 were loaded in the sample well on the device. After 10 min, the assay results were determined: negative (−), weak positive (±), or positive (+). Positive and weak positive results are considered CRP positive

A canine CRP ICA was developed by modifying a previously described technique [14]. Briefly, the kit was composed of a high-flow nitrocellulose membrane strip (MilliporeSigma, Burlington, MS, USA) prebound with the mAb 4D3C1 and anti-mouse IgG polyclonal antibodies (Southern Biotech, Birmingham, AL, USA) as a sample line and a control line, respectively, a glass filter strip (MilliporeSigma) absorbed with mAb 3B4D3 conjugated with colloidal golds, cellulose papers (MilliporeSigma), a backing sheet (DCN Diagnostics, Carlsbad, CA, USA), and a device case (DCN Diagnostics). The assay was performed by loading 5 μL of undiluted canine serum followed by 100 μL of BSA/PBS containing 0.1% Tween 20 in the sample well of the device. If both the red sample line and control line appeared in the test window of the device after 10 min, the assay was deemed to have a positive result, which was further distinguished as positive with a dark sample line and weak positive with a light sample line. If the sample line did not appear, the assay was deemed to have a negative result (Fig. 1).

The developed ELISA demonstrated an assay detection limit of 0.5 ng/mL, and a quantitative linear assay range of 1.6–100 ng/mL for the standard curve (R = 0.996) (Additional file 1). The intra-assay and inter-assay coefficient of variations were 0.7 to 10.0% and 6.0 to 9.0%, respectively, with a range of 3.2 to 50 ng/mL of CRP in serum samples diluted with BSA/PBS (n = 3) (Additional file 2). The spike tests in which three serum samples were spiked with known amounts of purified CRP demonstrated recovery rates ranging from 105 to 109% (Additional file 2). The parallelism assessments using four samples demonstrated recovery rates ranging from 82.7 to 104.4% in several sample dilutions (Additional file 3).

The canine serum test samples consisted of 16 samples from healthy blood donors as healthy control dogs (control group) and 38 samples from client-owned dogs presented to the Research Institute of Biosciences, Azabu University, Sagamihara, Japan as diseased dogs (patient group). All experimental protocols were approved by the Animal Experiment Committee of Azabu University.

**Fig. 2:**
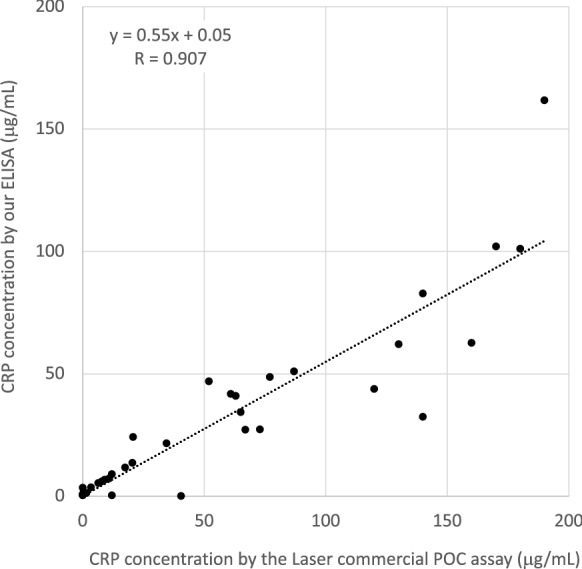
The correlation between the Laser commercial POC assay (x-axis) and our ELISA (y-axis) as seen with the assay results for 35 serum samples. The coefficient of correlation was 0.907 with the regression formula of y = 0.55x + 0.05

The CRP levels of the patient group were evaluated using both our ELISA and the Laser commercial POC assay at Azabu University (Additional file 4). Undiluted serum samples were applied to the Laser assay, but serum samples diluted to 1:1000 or higher with BSA/PBS were used for our ELISA because of its high assay sensitivity. Three of the 38 samples assayed were omitted from this analysis because their serum CRP levels were out of the Laser’s quantitative assay range. The correlation between the CRP levels obtained with our ELISA and those obtained with the Laser assay was 0.907 with the regression formula of y = 0.55x + 0.05 (Fig. 2). The slope of 0.55 suggests that our ELISA obtained approximately 55% lower CRP levels than the Laser assay. Since the Laser assay employs a CRP positive cut-off value of 10 µg/mL, our ELISA would have an equivalent positive cut-off value of 5.5 µg/mL. The difference may be due to the different standard preparation and validation methods of the two assays. Two sample results (sample #15 and #21) showed discrepancies between our ELISA and the Laser assay (Additional file 4). Generally, ELISAs tend to produce more reliable results than POC assays because of the ELISA’s multiple assay steps, such as washing steps. This may indicate that more investigation is required to obtain higher precise results with POC assays [13].

The mean value (± standard deviation: SD) of serum CRP assayed by our ELISA in the control group was 1.1 ± 1.3 µg/mL (range 0.1 to 4.6 µg/mL), which correlated well with the findings of previous reports [5–7]. While the mean value (± SD) of serum CRP in the patient group was 40 ± 53.4 μg/mL (range 0.1 to 259.3 µg/mL) (Additional file 5). The control group samples showed a lower range of serum CRP levels, while the patient group samples showed a very wide range of serum CRP levels. Interestingly, ten samples in the patient group had serum CRP levels lower than 4.6 µg/mL, at which point the range overlapped with the serum CRP levels in the control group. This indicated that the serum CRP level depends on the inflammatory condition in the individual dogs. However, it also suggested that serum CRP levels higher than those seen in healthy control dogs obviously indicate higher levels of inflammation. While the evaluation of serum CRP levels must be considered in the context of the clinical disease condition, we suggest that CRP levels assayed by our ELISA can distinguish noninflammatory and inflammatory conditions using approximately 5 µg/mL as the cut-off value (mean + 3SD: 1.1 + 3 × 1.3 μg/mL) based on the results for the control group, and equivalent cut-off values (5.5 µg/mL) were seen with the Laser assay in this study and in previous reports [11, 12].

**Fig. 3:**
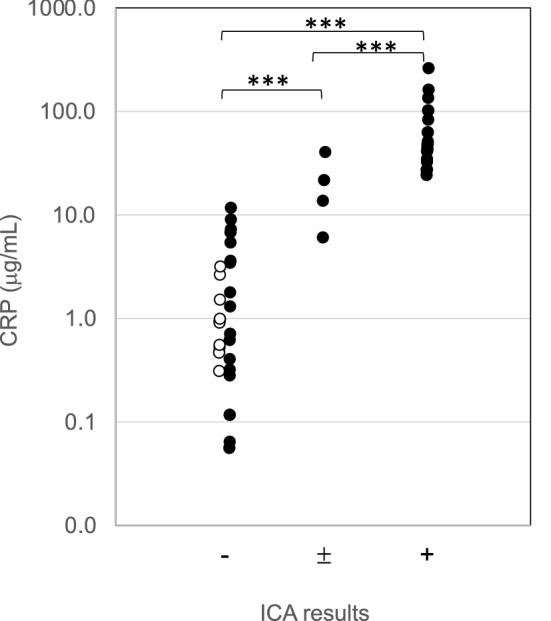
A scattergram of canine C-reactive protein (CRP) concentrations assayed by our ELISA (y-axis) against the results assayed by our ICA (x-axis) using 54 clinical samples (open circles: 16 samples in the control group; closed circles: 38 samples in the patient group) (***P < 0.001)

Canine serum samples with known CRP levels evaluated by our ELISA were assayed using our ICA. The ICA showed negative results for serum CRP levels lower than 11.7 µg/mL (mean ± SD: 2.3 ± 2.9 μg/mL) except for in one sample (6.0 μg/mL), weak positive results between 6.0 and 40.2 µg/mL (mean ± SD: 20.4 ± 14.7 μg/mL), and positive results for levels higher than 24.1 µg/mL (mean ± SD: 72.9 ± 59.0 μg/mL) (Fig. 3, Additional file 5). There were significant differences (P < 0.001) in serum CRP levels between the three individual assay results (Fig. 3). Because the ICA produced negative results for samples with a level lower than 11.7 µg/mL and weak positive results for samples with a level higher than 6.0 µg/mL, the cut-off value of the ICA was suggested to be approximately 5–10 µg/mL, which is comparable to the cut-off values of commercial assays [11, 12]. The cut-off value of the ICA was confirmed using diluted serum samples known CRP levels as approximately 5 µg/mL agreed with the results of clinical samples, producing negative results for samples with a level lower than 4.3 µg/mL and positive results for those with a level higher than 4.4 µg/mL (Table 1). Negative results in our ICA were obtained for the two samples (sample #15 and #21) with an observed discrepancy in results between our ELISA and the Laser assay and were agreed with our ELISA results (Additional files 4 and 5).

**Table 1:** Precision and accuracy tests of the canine CRP ICA using serial dilutions of four serum samples. The CRP levels were determined with our ELISA at a 1:1000 sample dilution. ICA results: −, negative; ± , weak positive; and +, positive

Sample #	Dilution	CRP (μg/mL)	ICA results
S1	1:2	38.2	+
	1:4	19.0	+
	1:8	9.3	+
	1:16	4.4	±
	1:32	2.0	−
	1:64	0.9	−
S2	1:2	29.4	+
	1:4	14.4	+
	1:8	7.0	±
	1:16	3.5	−
	1:32	1.6	−
S3	1:2	18.9	+
	1:4	9.1	±
	1:8	4.3	−
	1:16	2.1	−
S4	1:2	56.6	+
	1:4	29.2	+
	1:8	14.8	+
	1:16	6.9	±
	1:32	3.4	−
	1:64	1.7	−

These studies demonstrate that our ELISA shows good correlations with a commercially available POC assay and our ICA. The ICA can be used as an alternative diagnostic tool to evaluate the inflammation status in veterinary clinics that do not have analyzers.

In veterinary clinics, diagnostic kits commonly require blood, serum, and/or plasma test samples. However, unlike in human clinics, drawing blood from animals usually requires either restraints with a risk of animal bites for clinic workers or anesthesia with a risk for the patient. Alternatively, in human medicine, saliva and urine samples have been traditionally used for diagnostic test purposes. Saliva sample preparation is especially advantageous due to the collection method being noninvasive, nonstressful, and easy for patients.

Recently, it was reported that the CRP levels in saliva are approximately 1/100 levels of those in the serum, but salivary CRP levels correlate with serum CRP levels, and diseased dogs tend to express significantly higher salivary CRP levels than healthy dogs [9]. For detecting very low levels of salivary CRP, a higher sensitivity assay is required. This study suggests that our ELISA may be able to determine salivary CRP levels at an approximately 1:10 sample dilution instead of the 1:1000 dilution used for serum CRP analysis. The ICA was developed with the intent of reducing assay sensitivity during assay optimization to adjust an appropriate cut-off value for the serum CRP assay. Indeed, development of an ICA for saliva samples requires further assay condition optimization for determining ideal sample volumes, mAb concentrations, assay procedures, and assay time to achieve a sufficient assay sensitivity. We believe that a canine salivary CRP ICA would potentially be a convenient diagnostic tool in veterinary clinics.

Our newly developed canine CRP ELISA and ICA showed reliable assay results and a high correlation with a commercially available POC assay in clinical use. The ICA can be a useful canine CRP screening test for diagnostic purposes in veterinary clinics. In addition, the pair of mAbs used in our assays can be adapted for quantitative POC assays in the future.

## Supplementary information

**Additional file 1.** A standard curve for canine C-reactive protein (CRP) concentrations from 1.6 to 100 ng/mL generated with our one-step sandwich ELISA. Purified canine CRP was quantitated by using a combination of two mAbs: 4D3C1 as the capture antibody and 3B4D3 conjugated with horseradish peroxidase as the detection antibody.

**Additional file 2.** Precision tests using three CRP levels in our ELISA. Repeatability studies (intra- and inter-assay variations) using diluted serum samples with three CRP levels in BSA/PBS were performed in triplicate in each assay and on three different occasions. The spike tests were performed using three diluted serum samples spiked with known amounts of purified CRP. The results are expressed as the average recovery rates determined by dividing the observed CRP levels by the expected CRP levels.

**Additional file 3.** Parallelism assessments of our ELISA using four serially diluted serum samples. Each sample was serially diluted with BSA/PBS. The results are expressed as the recovery rates determined by dividing the observed CRP levels by the expected CRP levels.

**Additional file 4.** CRP levels of 38 samples in the patient group determined by our ELISA and the Laser commercial POC assay.

**Additional file 5.** CRP levels of 16 samples in the control group and 38 samples in the patient group determined by our ELISA and assay results for our ICA. ICA results; −: negative; ±: weak positive; +: positive.

## Data Availability

The datasets generated and analyzed during the current study are available from the corresponding author upon requests.

## Abbreviations

BSA: Bovine serum albumin
CRP: c-reactive protein
ELISA: Enzyme linked immunosorbent assay
HRP: Horseradish peroxidase
ICA: Immunochromatography test
mAbs: Monoclonal antibodies
POC: Point-of-care
