# Supplementary material for: Development of canine C-reactive protein assays
Source: Acta Vet Scand. 2020 Sep 7;62:50. doi: 10.1186/s13028-020-00549-9 (PMC7487759; doi:10.1186/s13028-020-00549-9)
Supplement: Supplementary file 1 — Additional file 1. A standard curve for canine C-reactive protein (CRP) concentrations from 1.6 to 100 ng/mL generated with our one-step sandwich ELISA. Purified canine CRP was quantitated by using a combination of two mAbs: 4D3C1 as the capture antibody and 3B4D3 conjugated with horseradish peroxidase as the detection antibody. [file 13028_2020_549_MOESM1_ESM.pdf]

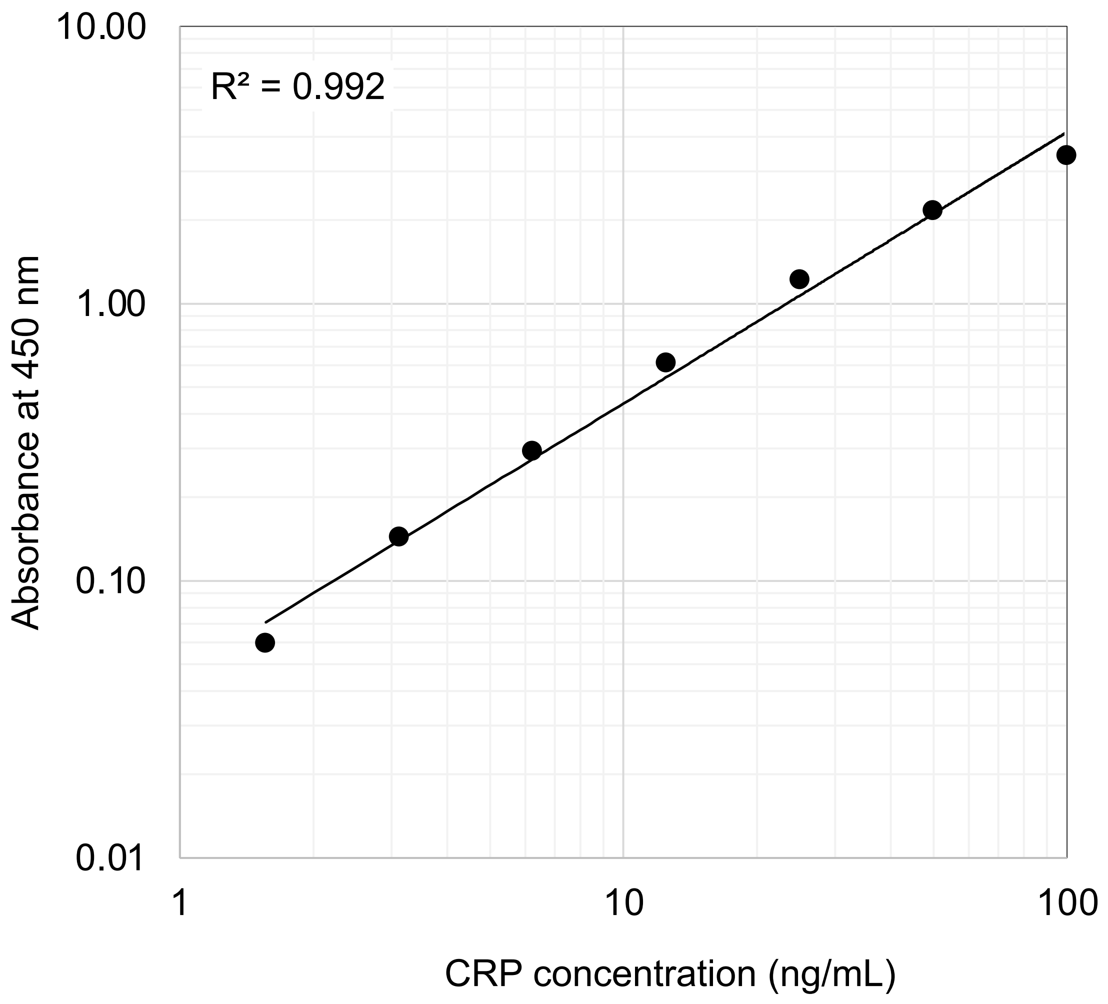

Additional file 1. A standard curve for canine C-reactive protein (CRP) concentrations from 1.6 to 100 ng/mL generated with our one-step sandwich ELISA. Purified canine CRP was quantitated by using a combination of two monoclonal antibodies: 4D3C1 as the capture antibody and 3B4D3 conjugated with horseradish peroxidase as the detection antibody.
