# Supplementary material for: Development of canine C-reactive protein assays
Source: Acta Vet Scand. 2020 Sep 7;62:50. doi: 10.1186/s13028-020-00549-9 (PMC7487759; doi:10.1186/s13028-020-00549-9)
Supplement: Supplementary file 2 — Additional file 2. Precision tests using three CRP levels in our ELISA. Repeatability studies (intra- and inter-assay variations) using diluted serum samples with three CRP levels in BSA/PBS were performed in triplicate in each assay and on three different occasions. The spike tests were performed using three diluted serum samples spiked with known amounts of purified CRP. The results are expressed as the average recovery rates determined by dividing the observed CRP levels by the expected CRP levels. [file 13028_2020_549_MOESM2_ESM.pdf]

Assays	CRP concentration (ng/mL)		
	3.2	12.5	50
Inter-assay CV (%)	6.0	9.0	8.7
Intra-assay CV (%)	1.0	10.0	0.7
Spike Test	108%	105%	109%

Additional file 2. Precision tests using three CRP levels in our ELISA. Repeatability studies (intra- and inter-assay variations) using diluted serum samples with three CRP levels in BSA/PBS were performed in triplicate in each assay and on three different occasions. The recovery study was performed using three diluted serum samples spiked with known amounts of purified CRP. The results are expressed as the average recovery rates determined by dividing the observed CRP levels by the expected CRP levels. CV:Coefficients of variations.
