# Supplementary material for: Development of canine C-reactive protein assays
Source: Acta Vet Scand. 2020 Sep 7;62:50. doi: 10.1186/s13028-020-00549-9 (PMC7487759; doi:10.1186/s13028-020-00549-9)
Supplement: Supplementary file 3 — Additional file 3. Parallelism assessments of our ELISA using four serially diluted serum samples. Each sample was serially diluted with BSA/PBS. The results are expressed as the recovery rates determined by dividing the observed CRP levels by the expected CRP levels. [file 13028_2020_549_MOESM3_ESM.pdf]

Sample #	Dilution	CRP concentration (ng/mL) Observed	Expected	% Recovery
S1	1:2000	38.2	38.2	100.1
	1:4000	19.0	19.1	99.7
	1:8000	9.3	9.6	97.1
	1:16000	4.4	4.8	91.2
	1:32000	2.0	2.4	82.7
S2	1:2000	29.4	29.4	100.1
	1:4000	14.4	14.7	97.7
	1:8000	7.0	7.4	95.6
	1:16000	3.5	3.7	94.9
	1:32000	1.6	1.8	88.4
S3	1:2000	18.9	18.9	100.2
	1:4000	9.1	9.5	96.5
	1:8000	4.3	4.7	90.3
	1:16000	2.1	2.4	90.8
S4	1:2000	56.6	56.6	99.9
	1:4000	29.2	28.3	103.3
	1:8000	14.8	14.2	104.4
	1:16000	6.9	7.1	97.4
	1:32000	3.4	3.5	95.2
	1:64000	1.7	1.8	93.6

Additional file 3. Parallelism Assessments of our ELISA using four serially diluted serum samples. Each sample was serially diluted with BSA/PBS. The results are expressed as recovery rates determined by dividing the observed CRP levels by the expected CRP levels.
