# Supplementary material for: Development of canine C-reactive protein assays
Source: Acta Vet Scand. 2020 Sep 7;62:50. doi: 10.1186/s13028-020-00549-9 (PMC7487759; doi:10.1186/s13028-020-00549-9)
Supplement: Supplementary file 4 — Additional file 4. CRP levels of 38 samples in the patient group determined by our ELISA and the Laser commercial POC assay. [file 13028_2020_549_MOESM4_ESM.pdf]

Sample #	CRP (μg/mL)	
	ELISA	Laser assay
21	0.1	40.5
15	0.3	12.0
3	0.3	0.1
2	0.4	0.1
4	0.6	0.1
5	0.7	0.1
7	1.3	1.5
6	1.8	0.5
1	3.4	0.1
8	3.6	3.5
9	5.4	6.5
10	6.0	8.0
11	6.7	9.0
13	7.2	11.0
14	9.0	12.0
17	11.7	17.5
18	13.6	20.5
20	21.6	34.5
19	24.1	20.7
27	27.1	67.0
28	27.3	73.0
37	32.4	140.0
26	34.3	65.0
46	40.2	200.0
25	41.0	63.0
24	41.7	61.0
34	43.9	120.0
23	47.0	52.0
29	48.7	77.0
30	50.9	87.0
35	62.1	130.0
38	62.7	160.0
36	82.8	140.0
40	101.1	180.0
39	102.1	170.0
42	134.8	>200
41	161.7	190.0
43	259.3	>200

Additional file 4. CRP levels of 38 samples in the patient group determined by our ELISA and the Laser commercial POC assay.
