# Supplementary material for: Development of canine C-reactive protein assays
Source: Acta Vet Scand. 2020 Sep 7;62:50. doi: 10.1186/s13028-020-00549-9 (PMC7487759; doi:10.1186/s13028-020-00549-9)
Supplement: Supplementary file 5 — Additional file 5. CRP levels of 16 samples in the control group and 38 samples in the patient group determined by our ELISA and assay results for our ICA. ICA results; −: negative; ±: weak positive; +: positive. [file 13028_2020_549_MOESM5_ESM.pdf]

Control Dogs		
Sample #	CRP (μg/mL)	ICA Result
54	0.1	-
58	0.1	-
61	0.1	-
50	0.3	-
51	0.5	-
53	0.5	-
52	0.5	-
62	0.5	-
47	0.9	-
48	0.9	-
57	1.0	-
55	1.0	-
49	1.5	-
60	2.6	-
59	3.1	-
56	4.6	-
Mean	1.1	
SD	1.3	

Patient Dogs		
Sample #	CRP (μg/mL)	ICA Result
21	0.1	-
15	0.3	-
3	0.3	-
2	0.4	-
4	0.6	-
5	0.7	-
7	1.3	-
6	1.8	-
1	3.4	-
8	3.6	-
9	5.4	-
10	6.0	±
11	6.7	-
13	7.2	-
14	9.0	-
17	11.7	-
18	13.6	±
20	21.6	±
19	24.1	+
27	27.1	+
28	27.3	+
37	32.4	+
26	34.3	+
46	40.2	±
25	41.0	+
24	41.7	+
34	43.9	+
23	47.0	+
29	48.7	+
30	50.9	+
35	62.1	+
38	62.7	+
36	82.8	+
40	101.1	+
39	102.1	+
42	134.8	+
41	161.7	+
43	259.3	+
Mean	40.0	
SD	53.4	

Additional file 5. CRP levels of 16 samples in the control group and 38 samples in the patient group determined by our ELISA and assay results for our ICA. ICA results; −: negative; ±: weak positive; +: positive. SD:standard deviation
